# Using the Da Vinci Single Port Robotic Platform in Abdominal Wall Surgery: Preclinical Exploration of a Suprapubic Single Port Extraperitoneal Approach (SP^2^ eTEP)

**DOI:** 10.3389/jaws.2025.15230

**Published:** 2025-10-03

**Authors:** Annabelle De Troyer, Francesco Brucchi, Filip Muysoms

**Affiliations:** ^1^ Department of General Surgery, Ziekenhuis Aan de Stroom (ZAS) Antwerp, Antwerp, Belgium; ^2^ Department of Pathophysiology and Transplantation, University of Milan, Milan, Italy; ^3^ Department of General Surgery, AZ Maria Middelares, Ghent, Belgium

**Keywords:** robotic surgery, single port, suprapubic eTEP, abdominal wall surgery, preclinical research

## Abstract

**Background:**

The Da Vinci Single Port (SP) robotic platform has recently been approved for general surgery in the European Union. However, its application in abdominal wall hernia repair remains largely unexplored. This study focuses on the development of a novel suprapubic single-port extraperitoneal approach (SP^2^ eTEP) for ventral hernia surgery.

**Method:**

Following the IDEAL framework for introducing new surgical procedures, this study details the preclinical exploration and technical development of SP^2^eTEP using the Da Vinci SP system. The research included procedural development on human cadavers and skills training using a porcine model.

**Results:**

Instrument reach and maneuverability were first evaluated using a silicone abdominal wall model. The model showed minimal reach loss between the 27 mm-SP metal cannula (29 cm straight, 27 cm articulated) and the collapsed SP small access port (28 cm straight, 26 cm articulated). Cadaveric sessions confirmed that the SP Access Port, placed suprapubically, allowed successful dissection in the preperitoneal, the retrorectus, and the subcutaneous planes. Additionally, bilateral component separation by transversus abdominis release was achieved using a bottom-up approach. The porcine model for inguinal hernia repair proved to be a suitable adjunct to simulator-based training for developing the necessary skills for subsequent clinical application.

**Conclusion:**

This preclinical (pre-IDEAL stage) exploration and procedural development demonstrate the feasibility of SP^2^ eTEP for ventral hernia repair with the Da Vinci SP platform. The findings support progression to clinical evaluation of this novel robotic approach for abdominal wall hernia repair.

## Introduction

Ventral hernia repair is a high-volume surgical procedure, increasingly performed using minimally invasive surgery (MIS) due to its association with reduced wound complications, faster recovery, and shorter hospital stays compared to open repair [1, 2]. Historically, laparoscopic intraperitoneal onlay mesh (IPOM) repair was the dominant MIS technique [3], but concerns about mesh-related complications—adhesions, bowel injury, and chronic pain from transfascial fixation—have led to a paradigm shift toward extraperitoneal mesh placement [4–7]. Recent evidence and international guidelines now favor sublay repairs with mesh placed in preperitoneal or retromuscular planes, which are associated with fewer complications and potentially improved long-term outcomes [1, 5, 8]. However, achieving these anatomical planes using conventional laparoscopy is technically demanding. Limitations in instrument articulation and visual angles, particularly for anterior wall dissection, have restricted adoption of extraperitoneal techniques such as transabdominal preperitoneal repair (TAPP), transabdominal retromuscular mesh repair (TARM/TARUP), extended totally extraperitoneal repair (eTEP), preperitoneal extended totally extraperitoneal technique (PeTEP), transversus abdominis release repair (TAR) and subcutaneous endoscopic onlay repair (SCOLA/ENDOR) [8–14]. The introduction of robotic platforms has significantly expanded the feasibility of complex MIS hernia repair [15, 16]. Robotic-assisted ventral hernia repair enables high-precision dissection, intracorporeal suturing, and advanced reconstruction techniques such as TAR via minimally invasive approaches [15, 17, 18]. These advancements have driven broader uptake of robotic extraperitoneal techniques, including robotic (P)eTEP and TAR, particularly in patients with midline or incisional defects [19–22]. Most robotic approaches to the abdominal wall currently rely on multi-port systems with lateral approaches. Recently, suprapubic approaches with cranial dissection have shown promise for accessing retrorectus and preperitoneal planes, offering ergonomic advantages and improved access to the lower midline [20, 23]. The Da Vinci Single Port (SP) platform—approved for general surgery in the European Union—introduces a compact, single-cannula system with flexible, wristed instruments that may further reduce tissue trauma and enable novel approaches to the anterior abdominal wall [24, 25].

To date, no systematic preclinical evaluation has assessed the feasibility of a suprapubic extraperitoneal approach using the Da Vinci SP system. In line with the IDEAL framework for procedural innovation [26], this study explores the preclinical development of a novel suprapubic single-port eTEP technique (SP^2^ eTEP) for abdominal wall surgery. The study evaluates anatomical reach, procedural feasibility, and skills acquisition in human cadaveric models and porcine live-tissue training, laying the groundwork for future clinical implementation.

## Methodology

**FIGURE 1:**
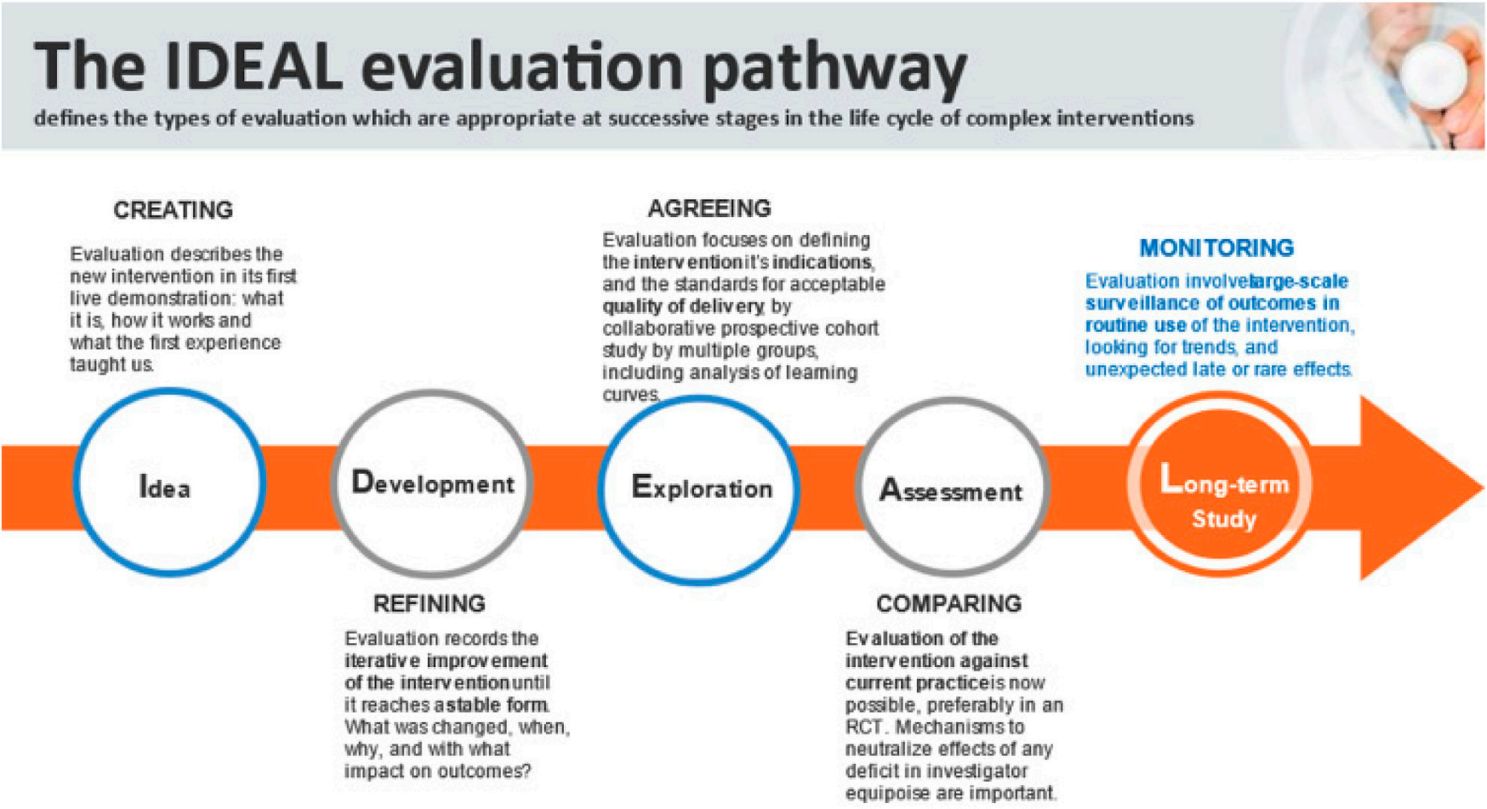
The IDEAL evaluation pathway for surgical innovation [26].

This preclinical study was designed in accordance with the IDEAL framework for surgical innovation, specifically addressing Stage 1 (Idea) and Stage 2a (Development) (Figure 1) [26]. The study consisted of three components [1]: dry-lab training and technical feasibility assessment [2], cadaveric procedural development, and [3] porcine model skills training.

### Dry-Lab Training and Technical Feasibility (IDEAL Stage 1: Idea)

Initial exploration of the SP robotic system was conducted with SimNow SP simulator training and with a dry-lab training environment, using a Dietz’ silicone abdominal wall model. The primary objective was to assess the range of motion (ROM) and reach of the SP instruments when introduced via the SP access port and metal cannula through a suprapubic trajectory. Reach to the upper midline (toward the xiphoid) was of particular interest, given its critical importance in enabling ventral hernia and diastasis repairs through a single low incision. The articulating capabilities of the SP system, as well as spatial constraints imposed by port placement, were systematically evaluated to inform subsequent procedural design.

### Cadaveric Procedural Development (IDEAL Stage 2a: Development)

A series of cadaveric dissections (n = 3) was undertaken to develop and refine the surgical steps of a SP^2^ eTEP approach. Dissections included preperitoneal and retrorectus plane development, midline plication for diastasis recti, and bilateral component separation using transversus abdominis release (TAR). Both suprapubic and transumbilical access points were explored to evaluate working angles, instrument triangulation, and the feasibility of completing a full bilateral TAR using the SP system.

### Porcine Model Skills Training (IDEAL Stage 2a: Development)

To consolidate SP-specific skills and validate procedural flow under live-tissue conditions, a structured training curriculum was implemented and performed using the previously described SPIRIT porcine model (n = 1) [27]. This model facilitates extraperitoneal dissection and mesh placement with anatomical and technical relevance to human abdominal wall surgery.

## Results

### Description of the DaVinci SP System

The Da Vinci SP Surgical System (Intuitive Surgical Inc., Sunnyvale, California) was used for all preclinical phases. It comprises a surgeon console with dual 3D-HD displays, a patient-side cart featuring a single robotic arm with four instrument drives (one 12 × 9 mm articulating endoscope and three 6-mm EndoWrist instruments), and a vision cart. All instruments are deployed through a 25 mm SP access port or metal cannula, enabling multi-directional manipulation and a nearly 360° working range within confined anatomical spaces [28].

### Description of the DaVinci SP Access Ports

The Da Vinci SP access port is a soft-tissue-compatible, balloon-based port designed to accommodate the SP robotic system’s single-arm architecture. It comprises a flexible introducer with a central lumen of approximately 25 mm, through which a multi-channel cannula is inserted.

The port features:

• An integrated balloon cuff, which is inflated once positioned to maintain port fixation and pneumopreperitoneum during surgery.
• A collapsible housing that, when deflated, enhances instrumental reach and range of motion.
• Low-profile external design, reducing the risk of arm-collision with patient limbs in caudal docking configurations.

In contrast, the rigid metal cannula is a non-collapsible alternative that provides slightly longer straight-line reach but reduced flexibility due to its fixed geometry. It may limit articulation in tight cranial regions unless sufficient working space is developed.

Both systems are compatible with the SP system’s Cobra camera mode, relocation function, and remote center control, which allow for dynamic adjustment of angles and positions during complex abdominal wall procedures.

### Dry-Lab Training and Technical Feasibility

The training pathway of robotic assisted surgery traditionally includes preclinic skills development using Da Vinci simulator training (SimNow). For the Da Vinci SP system similar SimNow models on the simulator are available to provide essential training on SP-specific features such as the Cobra camera mode and relocation function.

**FIGURE 2:**
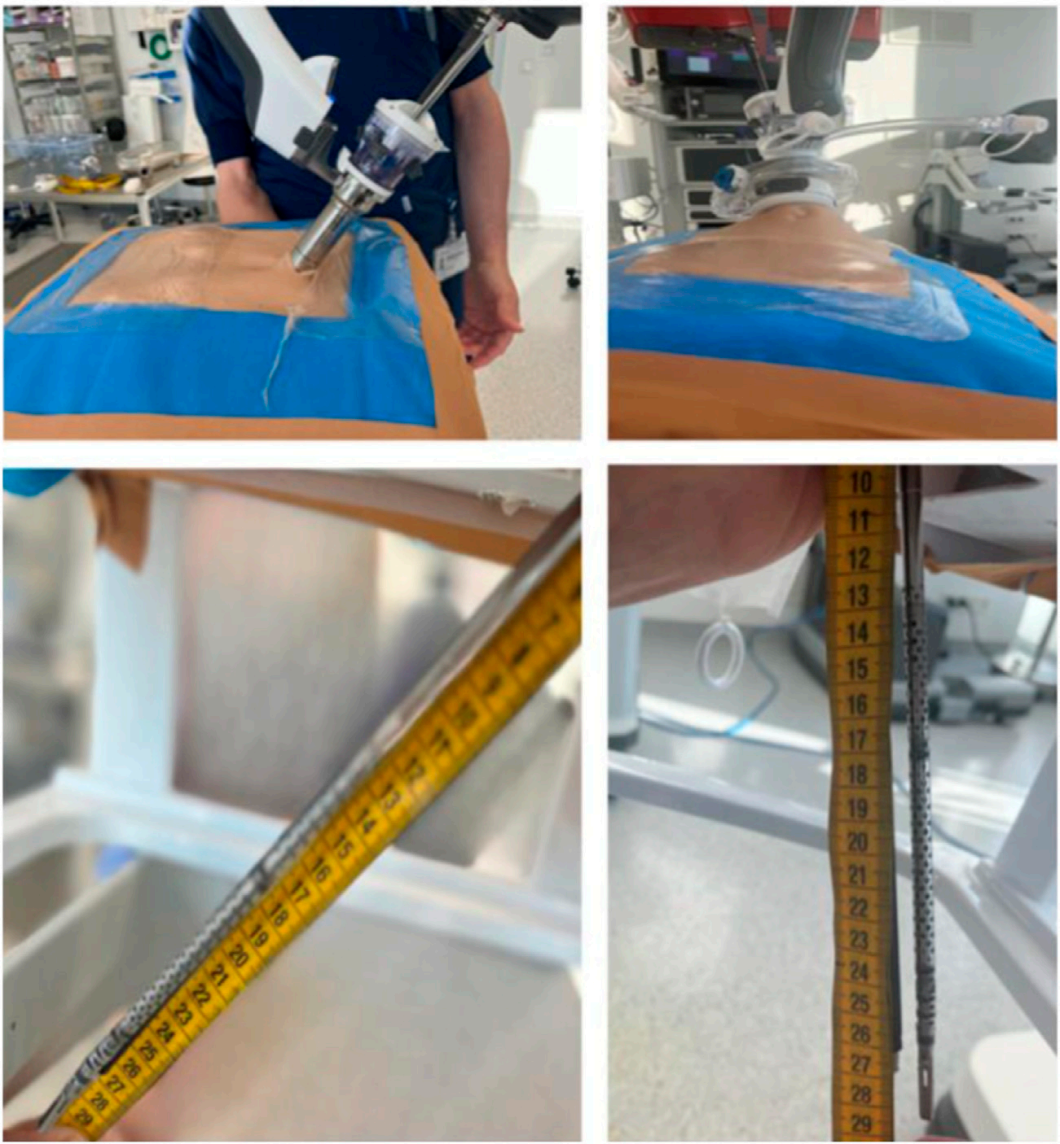
*Ex vivo* exploration performed using a Dietz’ silicone abdominal wall model to determine the ROM and reach of the instruments using the Da Vinci SP robotic system either with a metal cannula or with the SP small access port. (courtesy of Prof Ulrich Dietz from Olten, Switzerland).

In May 2025, a dry-lab testing using a Dietz’ silicone abdominal wall model to explore the effective reach and range of motion (ROM) of instruments in various configurations was conducted. Both the standard metal cannula and SP small access port were evaluated in their expanded and collapsed states (Figure 2). Maximum straight and articulated reach were measured from the skin to the instrument tip (Table 1).

**TABLE 1:** *Ex vivo* exploration performed using a Dietz’ silicone abdominal wall model to determine the ROM and reach of the instruments using the Da Vinci SP robotic system either with a metal cannula or with the SP small access port. (courtesy of Prof Ulrich Dietz from Olten, Switzerland).

Parameter	Overall instrument length	Metal Cannula	Access Port (Expanded)	Access Port (Collapsed)
Maximum Reach (cm) with straight instrument	*55*	*29*	*22*	*28*
Maximum Reach (cm) with Articulated instrument	*53*	*27*	*20*	*26*

### Cadaveric Procedural Development

#### Initial Lab (January 2022)

**FIGURE 3:**
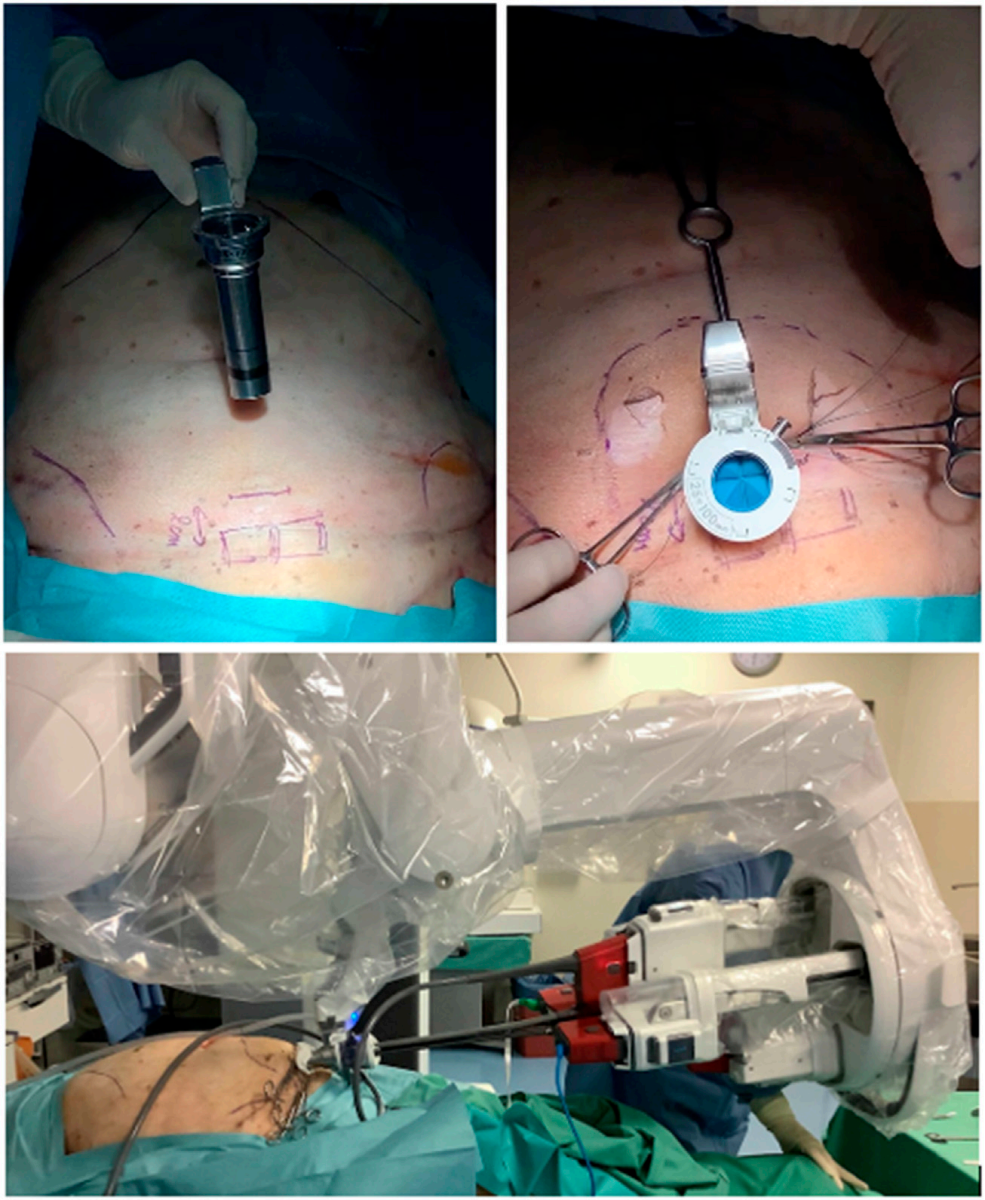
Cadaveric development lab using the metal SP cannula in a suprapubic position for an extraperitoneal retrorectus dissection (SP^2^ eTEP).

**FIGURE 4:**
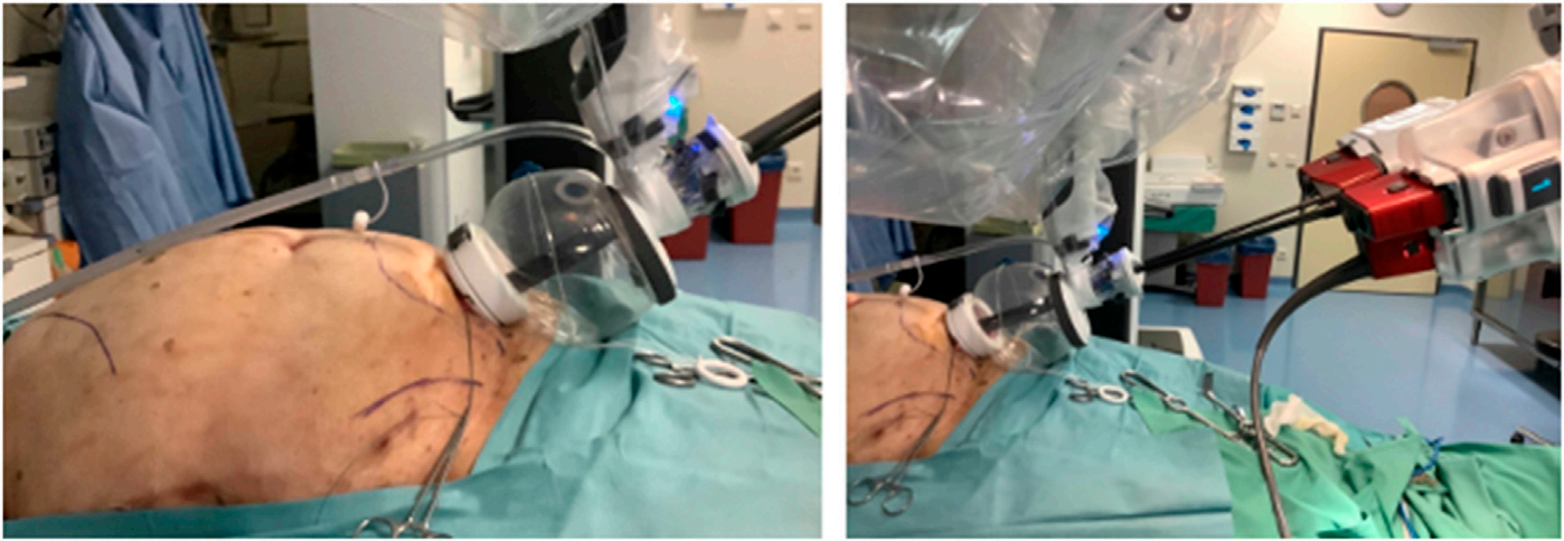
Cadaveric development lab using the SP small access port in a suprapubic position with a camera below position for an extraperitoneal retrorectus dissection (SP^2^ eTEP).

A procedure development lab was conducted to assess the feasibility of a suprapubic single-port extended totally extraperitoneal (SP^2^ eTEP) approach for ventral extraperitoneal abdominall wall surgery. The cadaver was placed in dorsal decubitus with the table flexed 15° and a head-down tilt. A 2.7 cm horizontal incision was made 2 cm above the pubic bone to access the preperitoneal space via a “mini-Pfannenstiel” technique. Blunt dissection enabled creation of a preperitoneal working space to introduce the metal SP cannula or small access port (Figure 3). Initial exploration using the metal SP cannula allowed successful dissection of the retrorectus space. However, articulation of the instrument elbows was limited until space was developed cranially. Swapping to the SP small access port improved articulation and maneuverability, especially in the cranial third of the retrorectus dissection. Switching between “camera above” and “camera below” views demonstrated that “camera below” in Cobra mode was optimal for anterior wall procedures (Figure 4).

**FIGURE 5:**
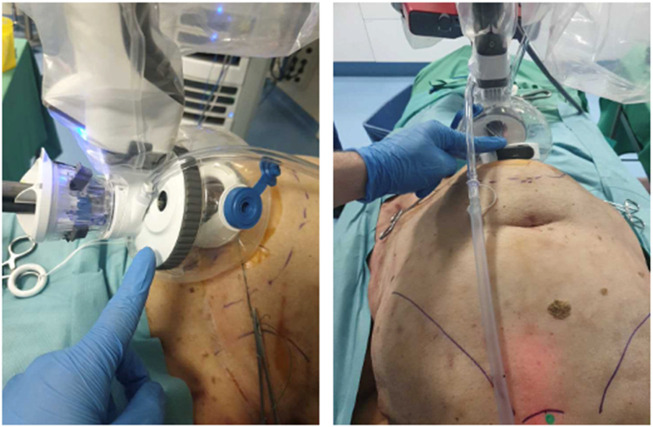
Cadaveric development lab using the SP small access port in a suprapubic position for extraperitoneal retrorectus dissection (SP^2^ eTEP). The access port is collapsed for maximal ROM and instrument reach cranially.

**FIGURE 6:**
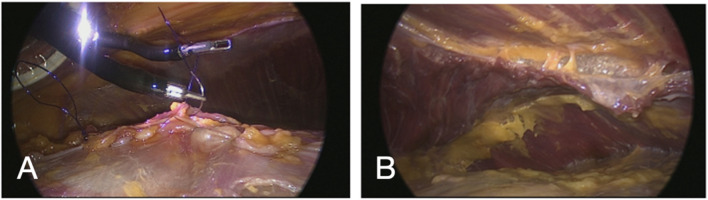
Laparoscopic view with a regular laparoscope through an extra observation trocar during a cadaveric development lab using the SP small access port in a suprapubic position for extraperitoneal retrorectus dissection (SP^2^ eTEP). Suturing posterior fascia **(A)** and view of TAR dissection achieved with dissection behind the diaphragm above the watershed fat **(B)**.

To increase reach near the xiphoid during midline diastastis closure and TAR near the diafragm, the balloon of the SP access port was collapsed, yielding improved access without trocar displacement (Figure 5). Suturing of the posterior rectus sheath and TAR near the diaphragm were feasible (Figure 6). Mesh insertion was easily performed via the SP assist port.

#### Follow-Up Lab (January 2024)

Following CE mark approval for general surgery in Europe, a second cadaveric lab tested software upgrades including the customized remote center (CRC) and pitch limit features. These allowed for safer manipulation without a metal trocar and minimized risk of arm collision with the patient’s lower extremities. The retrorectus dissection, midline closure, and mesh placement were successfully repeated.

**FIGURE 7:**
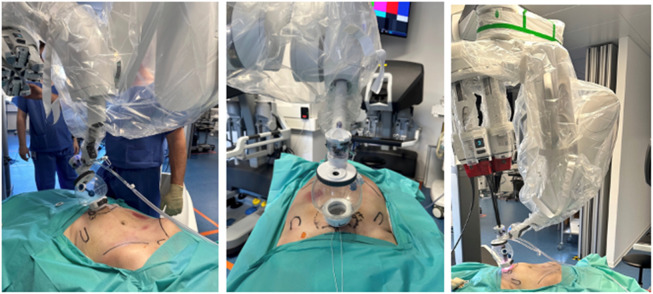
Cadaveric development lab using the SP small access port in a suprapubic position with a camera below position for extraperitoneal retrorectus dissection (SP^2^ eTEP).

**FIGURE 8:**
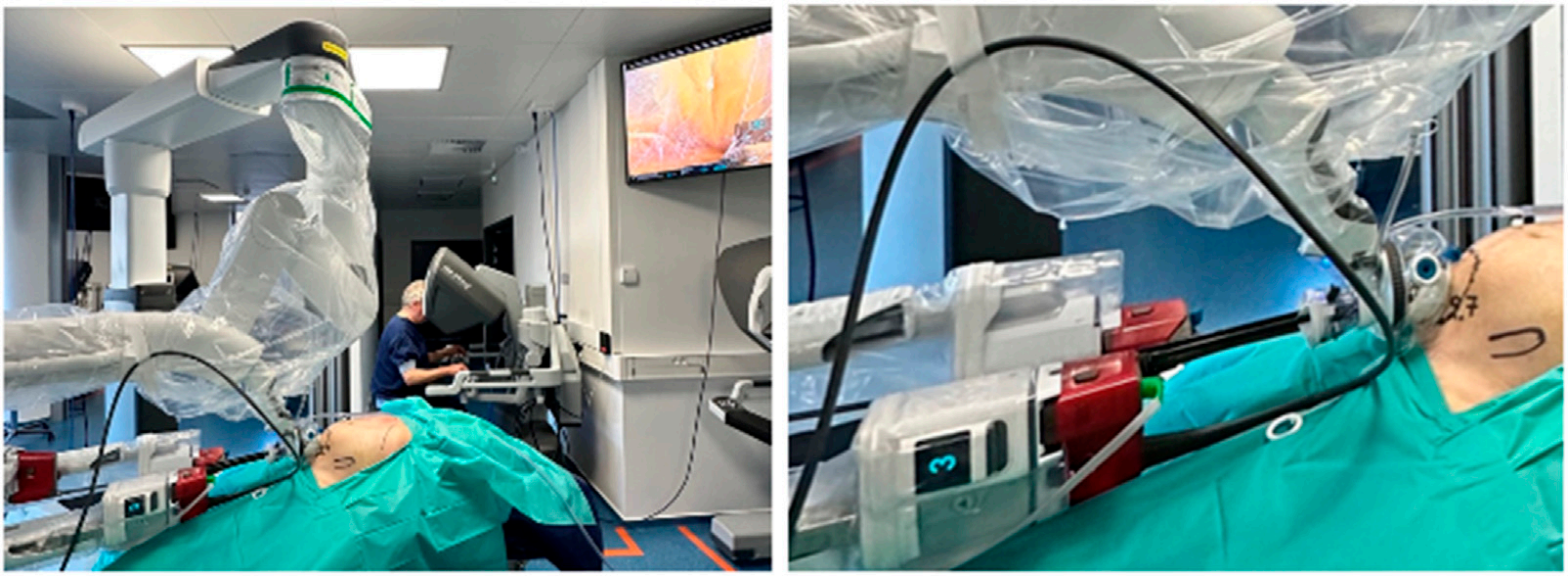
Cadaveric development lab using the SP small access port in a suprapubic position with a camera below position and “collapsed Access Port Balloon” working close to the Pitch limit during the most cranial part of the extraperitoneal retrorectus dissection (SP^2^ eTEP).

To further test system flexibility, the SP access port was relocated to the umbilicus, allowing a successful bilateral TAR without redocking, thanks to the relocation function and nearly 360° workspace (Figures 7, 8).

### Porcine Model Skills Training

**FIGURE 9:**
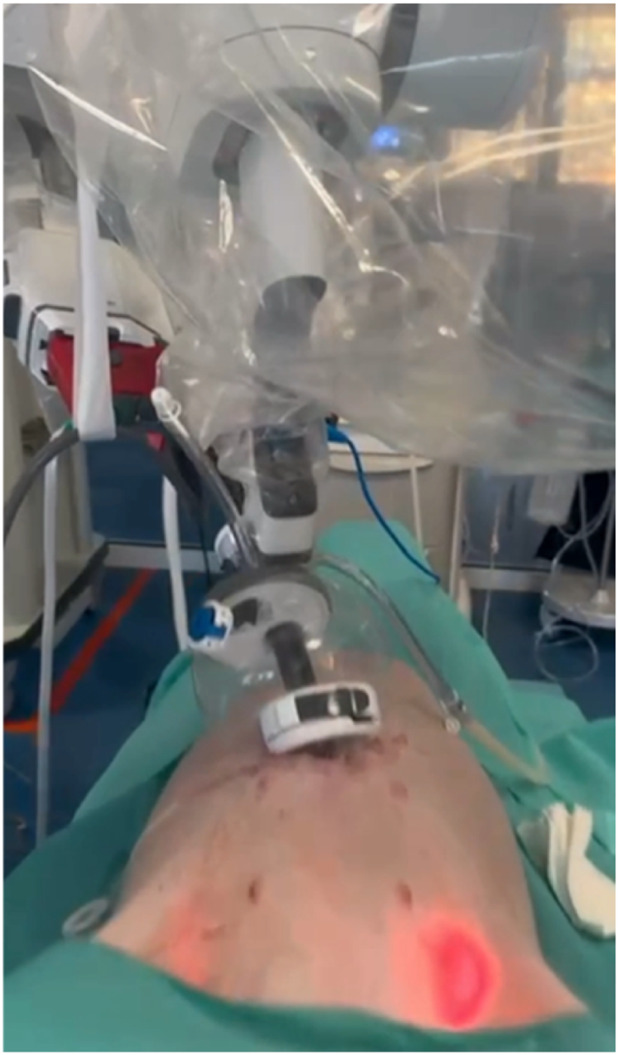
Porcine model skills development using the Da Vinci SP system and the SP small access port for a transabdominal preperitoneal mesh repair in the groin using to the SPIRIT model.

To complement simulator-based SP system training (SimNow), a skills development lab was conducted using the SPIRIT porcine model. This live-tissue model on anesthetized pigs, included in the Da Vinci’s TR300 training curriculum for DaVinci X en Xi system, facilitates extraperitoneal dissection, suturing and mesh placement with anatomical and technical relevance to human abdominal wall surgery [27]. Prior to the clinical use of the Da Vinci SP system, a porcine model skills training lab was performed, exploring the specific features of the SP system while performing a transabdominal preperitoneal repair in the groin (Figure 9). No intraoperative or postoperative complications were observed during the porcine model procedures. As per the standardized training protocol, animal were euthanized under general anesthesia at the end of the session, in full compliance with ethical guidelines.

### Cadaveric Standardization and SCOLA Exploration (March–April 2025)

**FIGURE 10:**
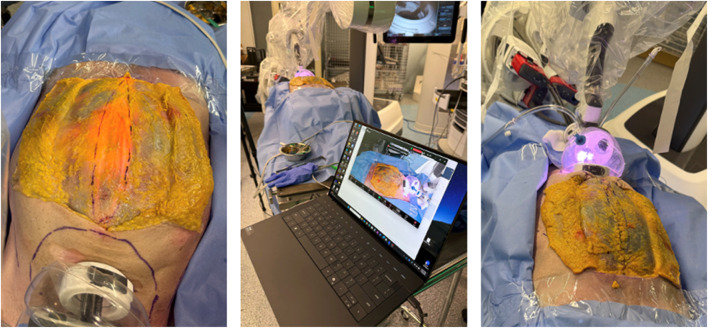
Cadaveric development lab using the SP small access port in a suprapubic position for extraperitoneal retrorectus dissection (SP^2^ eTEP). The skin and subcutaneous fat has been dissected to expose the anterior abdominal wall and visualize the diastasis plication using the “Inan stitch”.

Based on this experience, a procedural standardization lab was conducted in April 2025. In addition to SP^2^ eTEP, we explored the feasibility of performing a SubCutaneous OnLay endoscopic Approach (SCOLA) for diastasis and onlay mesh repair using the SP system. The SP small access port allowed stable maintenance of the subcutaneous working space without leakage. Dissection was followed by a preperitoneal approach and plication of the diastasis using barbed sutures in an Inan inverting (Connell-type) fashion, avoiding midline bulging post-repair (Figure 10).

These results collectively validated the SP^2^ eTEP technique as feasible and adaptable, and identified the SP system as a promising platform for single-incision ventral abdominal wall surgery.

## Discussion

This preclinical study aimed to explore and standardize the use of the Da Vinci SP robotic system for ventral abdominal wall surgery through a suprapubic approach—termed the SP^2^ eTEP technique. In alignment with IDEAL stage 1 (Idea) and stage 2 (Development) [26], we evaluated system capabilities, instrument reach, procedural steps, and training pathways necessary for safe clinical implementation.

Our dry-lab assessments and cadaveric procedure development sessions demonstrated that the Da Vinci SP platform provides sufficient range of motion and anatomical access to perform simple and complex ventral hernia repairs, including retrorectus mesh placements, preperitoneal dissections, and subcutaneous (SCOLA/ENDOR) reconstructions. The system’s single-arm architecture and fully articulating instruments allowed nearly 360° access, critical for reaching the xiphoid region through a single suprapubic incision. One of the main challenges during the initial cadaveric development of the SP2 eTEP technique was the limited working space available at the beginning of the dissection, especially when using the rigid metal cannula. Due to the confined preperitoneal environment, there was insufficient room for proper deployment of the SP instruments, significantly impairing triangulation and range of motion during the early steps of retrorectus dissection.

One major insight was the performance benefit offered by the SP small access port compared to the traditional metal cannula. The access port’s collapsibility enabled improved reach during cranial dissections and midline closure near the xiphoid. The reach of the collapsed acces port approximated the reach of the metal cannula while retaining full articulation (Table 1; Figure 2 summarize these findings.) This adaptability was especially important in completing complex reconstructions such as bilateral TAR, which was successfully performed from a centralized umbilical access point.

Skills acquisition using a structured simulation pathway is crucial for robotic proficiency. In our training program, we combined SP-specific simulator modules (SimNow) with the SPIRIT porcine model, a validated platform for robotic TAPP procedures [27]. This hybrid model effectively prepares surgeons for both the unique controls of the SP system and the anatomical subtleties of extraperitoneal dissection.

In anticipation of clinical use, we carefully selected early patient cases involving inguinal hernias with concomitant umbilical defects and ventral hernias with diastasis. This strategy aligns with EHS recommendations to adopt straightforward index procedures when implementing new robotic platforms. Furthermore, this diminishes the risk for port-site incisional hernias, which is a key concern after single-port techniques. In the suprapubic approach, we used a mini-Pfannenstiel incision known for its low hernia recurrence rate, and we extended mesh coverage caudally to mitigate the risk of port-site hernias.

We acknowledge that one of the key concerns with new robotic techniques is the potential for increased cost. Further investigation is warranted to compare the overall cost-effectiveness of SP versus multiport robotic and laparoscopic hernia repair, particularly considering operative time, recovery, and complication rates.

Overall, our findings provide a strong foundation for clinical translation of the SP^2^ eTEP approach. This article marks the completion of our preclinical IDEAL phase, and it serves as the basis for an upcoming prospective case series evaluating safety, reproducibility, and short-term outcomes of ventral hernia repair using the Da Vinci SP system. We believe this next step is essential to validate the clinical utility of the SP2 eTEP technique and refine its indications for broader adoption.

## Data Availability

The raw data supporting the conclusions of this article will be made available by the authors, without undue reservation.
